# Wellbeing and retainment of healthcare workers in Wales from 2020 to 2023

**DOI:** 10.23889/ijpds.v9i5.2757

**Published:** 2024-09-18

**Authors:** Stuart Bedston, Hoda Abbasizanjani, Lucy Robinson, Matthew Curds, Ashley Akbari

**Affiliations:** 1 Swansea University; 2 Welsh Government

## Objectives

The COVID-19 pandemic greatly impacted the wellbeing of healthcare workers (HCWs) due to stress, longer hours, and risks of infection during an uncertain time. Post-pandemic, we need to understand the extent of the lasting impacts on wellbeing and staff retainment.

## Approach

Using linked administrative health and workforce records within the SAIL Databank, we conducted a cohort study of HCWs employed directly by the National Health Service in Wales between 2020 and 2023. We investigated trends in HCW turnover by fiscal quarter and staff group and the prevalence of occupational health issues per primary care records, including authorised sick leave. We analysed time until leaving the profession using Cox regression incorporating workforce, demographic and health characteristics.

## Results

From January 2020 to December 2023, HCW employment grew from 101,600 to 121,970 (+20.0%). During this period, 34,760 people left the profession, averaging a turnover of 4.4% per fiscal quarter. Equally, an average of 1.9% of HCWs were diagnosed with a mental health issue, and 17.9% received a related prescription per fiscal quarter. We present further results highlighting how staff turnover has increased over time and differed across staff groups. We also show the extent to which leaving the profession is associated with workforce, demographic and health characteristics.

## Conclusion

The rate of mental health issues and increases in turnover from the profession highlight the need for additional support. Healthcare systems need to prioritise the welfare of their workforce to ensure sustained resilience and quality of care.

